# *Leonhard Med*, a trusted research environment for processing sensitive research data

**DOI:** 10.1515/jib-2024-0021

**Published:** 2024-08-02

**Authors:** Michal J. Okoniewski, Anna Wiegand, Diana Coman Schmid, Christian Bolliger, Cristian Bovino, Mattia Belluco, Thomas Wüst, Olivier Byrde, Sergio Maffioletti, Bernd Rinn

**Affiliations:** SIS Scientific IT Services, ETH Zurich, Binzmühlestrasse 130, 8092 Zurich, Switzerland, https://sis.id.ethz.ch/

**Keywords:** trusted research environments, high performance computing, secure data processing, personalized medicine, human bioinformatics, cross-omics research platforms

## Abstract

This paper provides an overview of the development and operation of the *Leonhard Med* Trusted Research Environment (TRE) at ETH Zurich. *Leonhard Med* gives scientific researchers the ability to securely work on sensitive research data. We give an overview of the user perspective, the legal framework for processing sensitive data, design history, current status, and operations. *Leonhard Med* is an efficient, highly secure Trusted Research Environment for data processing, hosted at ETH Zurich and operated by the Scientific IT Services (SIS) of ETH. It provides a full stack of security controls that allow researchers to store, access, manage, and process sensitive data according to Swiss legislation and ETH Zurich Data Protection policies. In addition, *Leonhard Med* fulfills the BioMedIT Information Security Policies and is compatible with international data protection laws and therefore can be utilized within the scope of national and international collaboration research projects. Initially designed as a “bare-metal” High-Performance Computing (HPC) platform to achieve maximum performance, *Leonhard Med* was later re-designed as a virtualized, private cloud platform to offer more flexibility to its customers. Sensitive data can be analyzed in secure, segregated spaces called tenants. Technical and Organizational Measures (TOMs) are in place to assure the confidentiality, integrity, and availability of sensitive data. At the same time, *Leonhard Med* ensures broad access to cutting-edge research software, especially for the analysis of human -omics data and other personalized health applications.

## 1 Introduction

In numerous scientific research fields, managing sensitive data is crucial. A prominent example of this necessity is seen in handling patient data within personalized health research. Stringent legislation ensures the protection of such information. In Switzerland, the update of the Federal Act on Data Protection (FADP) [1] in September 2023 has brought additional requirements for the safeguarding of sensitive data, such as maintaining a registry detailing processing activities. Moreover, federal entities must log all automated processing operations involving such data. Additionally, for the handling of data pertaining to persons (study participants or patients), biological material, or health-related personal data the Federal Act on Research Involving Human Beings (HRA) [2] applies as an additional regulatory framework. On top of that researchers are subject to institute-specific guidelines for handling sensitive data. It is noteworthy that *Leonhard Med* complies with the aforementioned legislations.

To meet the needs of the research community for processing sensitive data securely and according to legislation, ETH Zurich’s Scientific IT Services (SIS) have developed, implemented, and are operating the *Leonhard Med* Trusted Research Environment (TRE). Tailored with personalized health as its central focus, *Leonhard Med* is structured to accommodate a diverse range of sensitive data, including historical or political data, alongside other categories. Thanks to its participation to the *Swiss Personalized Health Network* (SPHN) program, *Leonhard Med* is available to all researchers of any Swiss academic institution.

*Leonhard Med* is a secure, powerful and versatile TRE to transfer, store, manage and analyze sensitive research data. At the national level, SIS with the *Leonhard Med* TRE is part of the BioMedIT network, representing the node Zurich. BioMedIT is a secure IT network open to all Swiss universities, research institutes, hospitals, service providers and other interested partners. The project is lead by the Swiss Institute of Bioinformatics (SIB) and tightly coordinated with the *Swiss Personalized Health Network* (SPHN) [3], [4], [5], [6] and the *Personalized Health and Related Technologies* (PHRT) program of the ETH Domain. BioMedIT facilitates researchers’ work by offering end-to-end secure data handling, remote access to project spaces via cloud technology, collaborative data processing, and ensures compliance with regulatory requirements while providing ongoing support throughout the research projects. The BioMedIT central services include process support for the execution of large, multi-center health-related research projects. Through the BioMedIT portal and tools it assures data transfer of sensitive data between different data owners and BioMedIT TREs. Interoperability of multi-center projects (such as [7]) includes other challenges like harmonization of semantic annotations of clinical data. This central perspective and the related challenges are relevant in establishing personalized health research in Switzerland.

*Leonhard Med* was conceived and constructed in response to the needs of the research community, predating the surge of discussions in research and engineering journals. Currently, research studies on TREs are ongoing, but still without a particular consensus on the major design features of such environments [8], [9], [10], [11]. Still, in most of the cases, at least some distinctive guidelines similar to those described in the conclusions of Ref. [8] are being put into practice in academic and medical institutions. In addition, to the best of our knowledge, the literature is very sparse regarding similar use cases with systematic descriptions and documentation. The systems most similar to *Leonhard Med* at present appear to be the *Secure Research Data and Computing* (SRDC) system developed by the Research IT team at the University of California, Berkeley [12], eMedLab of Sanger and the epouta system implemented by the Finnish IT Center for Science (CSC) [13]. Therefore, the primary motivation for this paper is to share our knowledge and experiences in constructing a large-scale, multi-user TRE for handling sensitive data with the interested community.

## 2 History of the *Leonhard Med* trusted research environment

The *Leonhard Med* TRE has been meticulously planned and designed by the Scientific IT Services of ETH Zurich (SIS) since 2016, running in tandem with the implementation of the Swiss Personalized Health Network (SPHN) program [5]. Initially, its primary objective was to furnish secure data processing capabilities to projects funded through SPHN grants and the use cases of specific ETH researchers. Consequently, the design specifications were extensively deliberated upon with the prospective ETH user base, enabling researchers to analyse those data with cutting edge computing and software resources in a TRE.

In the beginning, the main user requirement was that the TRE needs to be similar in usage to the established and well-known HPC platforms of ETH Zurich (such as the Euler cluster [14]), because the main part of the work on sensitive data was expected to be computational biology data with large compute demands and typically, such bioinformatic workflows are developed and tested on standard HPC platforms with public datasets and later ported to a secure cluster for the production phase on sensitive data. Later, the use cases became more diverse and *Leonhard Med* was expanded to cover data management solutions, custom applications and services like web-based applications, and integration with existing community services and initiatives. Additional requirements that are equally important for a TRE like *Leonhard Med* include:

– High level of security, akin to banking applications, also following the ETH and SPHN security guidelines [15, 16].
– Technical measures for access restriction: Strict separation between data from various projects and research groups, going beyond standard POSIX access usually applied in an HPC environment.
– Organizational measures for access restriction: User authorization is facilitated via request to the *Leonhard Med* Service Desk and a regular review of user lists by the Project Leader is enforced.
– Possibility to run community-specific services and tools to accommodate the needs of a specific research group.
– Integration with existing external sensitive data management platforms and services, both at the national and international level.
– Harmonisation of sensitive data transfer from a large variety of existing data sources like university hospitals, genome centers with systems such as described in [17, 18]. Providing end-users with a common experience on data transfer while integrating different sources with different requirements and in some cases, different policies and technical means.
– Despite the constraints of high security mechanisms, researchers must be able to download and install the software of their choice, such as the newest versions of bioinformatics tools or specific novel code repositories from github.

**Figure 1:**
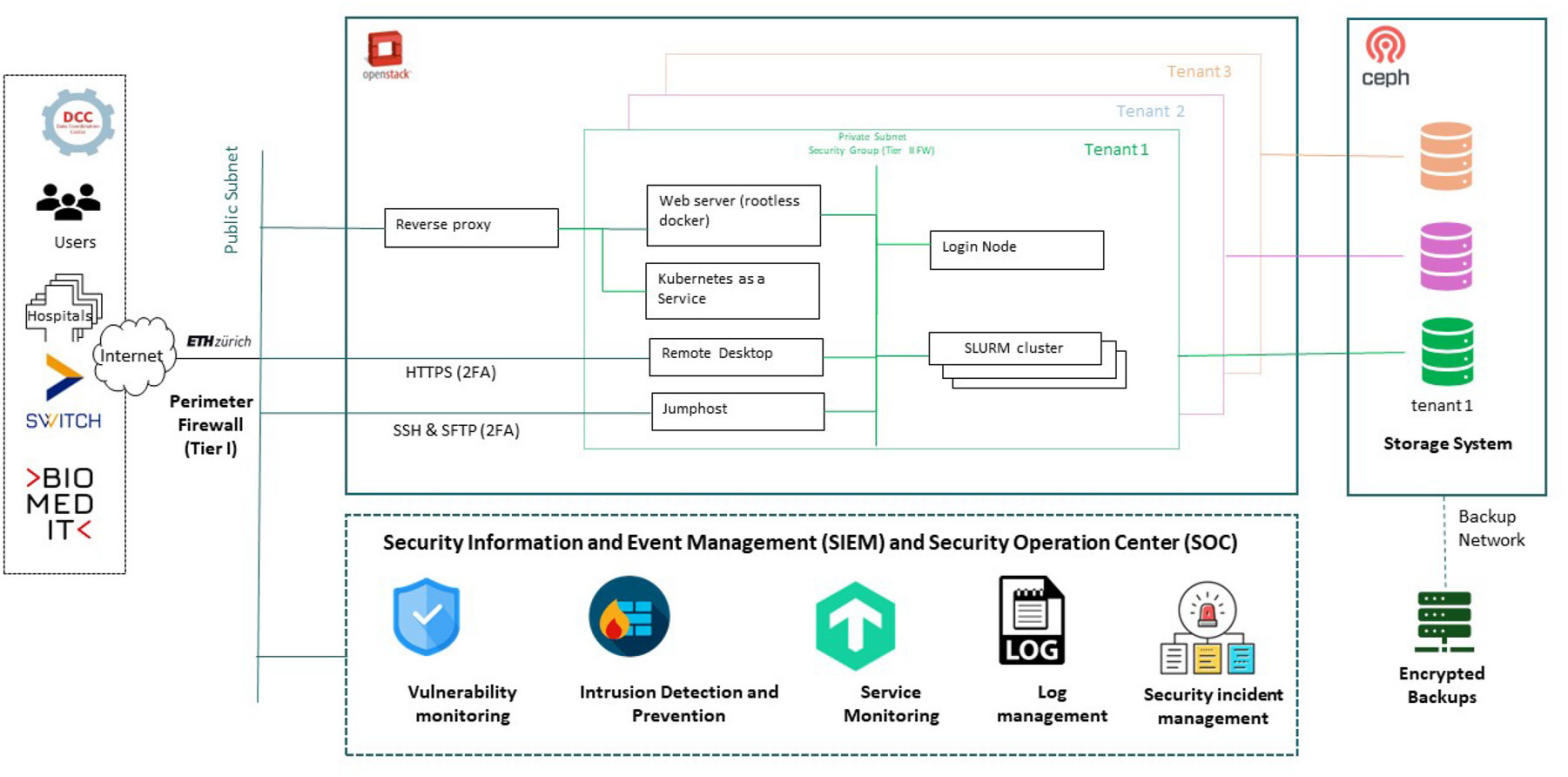
*Leonhard Med* architecture overview.

The mentioned requirements were integrated into the initial release of *Leonhard Med* in 2017. The TRE has since undergone ongoing enhancements, resulting in a significant upgrade in 2023 (referred to as *Leonhard Med* 2.0), where the system was transformed into a cloud-based environment, emphasizing customization, and resource sharing, as illustrated in Figure 1.

## 3 Customers and tenancy system

*Leonhard Med* customers benefit from the in-house control of the TRE in various ways, for example, the continuous development and the possibility to tailor the platform to user needs. The personal contact to the *Leonhard Med* team is something that distinguishes the TRE from commercial solutions.

**Figure 2:**
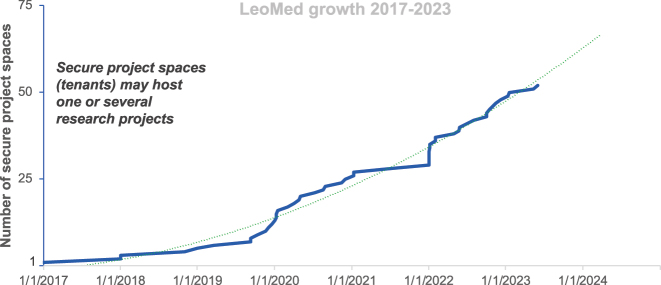
*Leonhard Med* growth in terms of number of tenants.

The research groups and project consortia can use the *Leonhard Med* TRE by purchasing a share of its infrastructure resources (compute, storage) and services, paid typically for 1 or 4 years. Each research group receives a secure tenant which is dedicated to a single Project Leader who may use it for one or several research projects A tenant consists of a private network space containing access, computing and data resources and it is protected by different security layers. A tenant is an ensemble of resources (login, compute nodes, storage, network) in *Leonhard Med* with its own security policies. The growing number of tenants in is depicted in the Figure 2.

There are two main types of tenants in *Leonhard Med*. The **Secure Isolated Tenant** has security level 2 (according to levels of IEC 62443-3-3), meaning a very high security with respect to normal research IT infrastructures. All the tenant resources (i.e., data, user access, computing, and software applications) are completely separated from other tenants in *Leonhard Med* by logical network isolation. In a **Secure Shared Tenant**, that assures Security level 1, the data within the tenant allocation are separated from data belonging to other allocations by UNIX file permission rules. In both tenant types all security controls of *Leonhard Med* are enabled.

*Leonhard Med* is an ensemble of resources that can be used by multiple tenants at the same time, while ensuring that users of two tenants cannot access each other’s data at any time. To guarantee the separation between its customers, *Leonhard Med* is a multi-tenant system built on top of an Openstack private cloud.

The general Service Level Agreement with SIS is a prerequisite for becoming a *Leonhard Med* customer. In addition, the Project Leader and all users within each tenant must sign the Acceptable Use Policy (AUP) of *Leonhard Med* [19]. This document defines the roles and responsibilities for User, Project Leader, and Permissions Manager, as well as the special usage rules applicable to *Leonhard Med*.

## 4 Extension of HPC platform towards cloud-based use cases

The *Leonhard Med* platform has evolved from the idea of a secure HPC cluster with login nodes, high-performance compute nodes and storage, where all computing resources are under the control of the Slurm scheduler [20]. Such a setup is appealing to bioinformaticians who develop and run their workflows in HPC environments. On *Leonhard Med* they can port the workflows directly into the secure environment and apply to the sensitive, e.g. medical data.

When logging in to *Leonhard Med*, a user has access to a tenant’s login node. As in standard HPC systems, login nodes have limited computing resources and should only be used to organize files and for simple pre- and post-processing tasks. Compute-intensive jobs are submitted to the tenant’s dedicated Slurm scheduler and executed on the tenant’s compute nodes.

Common use cases at *Leonhard Med* for working with sensitive data include the following scenarios:

– Analyzing sensitive data on a high-performance compute cluster.
– Hosting a web application that aggregates and plots sensitive data.
– Managing sensitive data using a data management solution, such as Ref. [21].
– Sharing sensitive data with a collaborating research group.
– Working with sensitive data from multiple Swiss hospitals.
– Storing sensitive data.

Those examples of scenarios can be customized on request by the *Leonhard Med* team, but they go beyond the standard HPC setup. To enable more versatile solution, *Leonhard Med* has evolved towards a cloud-based platform with extensive deployment and use of customized virtual machines (VMs).

*Leonhard Med* provides a large variety of virtualized nodes ranging from general purpose compute nodes to high-end GPU nodes. The current set configuration is publicly available form of a price list on the website.

## 5 Security mechanisms

*Leonhard Med* is designed with data security and usability as primary principles, and it implements a variety of mechanisms to achieve these objectives. The tenancy separation mechanism described above in Chapter 3 is one of those mechanisms. There are many other hardware, software and organizational aspects intended to ensure the security level appropriate for sensitive research data. The researchers benefit from “security as a service” allowing them to focus on their research without having to implement the security mechanisms themselves.

### 5.1 Physical security

The data center hosting *Leonhard Med* is located in an ETH building in Zurich, Switzerland. Two additional locations in Zurich host the systems where the backups are periodically sent.

The *Leonhard Med* operation team is responsible for the physical infrastructure that is hosted inside the data center. Access to the *Leonhard Med* secured server room is allowed exclusively to authorized personnel. In emergency situations, the ETH Safety and Security staff may access by intervention key the *Leonhard Med* server room. Access to both the ETH server room and the dedicated *Leonhard Med* room is logged, with logs kept over a period of three months. The *Leonhard Med* server room is fully protected against power failures. The uninterruptible power supply (UPS) protects the servers against short power losses or spikes, but not against longer interruptions.

### 5.2 Middleware security

*Leonhard Med* middleware is an ETH Zurich self-hosted private cloud that offers the baseline infrastructure for provisioning of virtualized network, compute, and storage. All *Leonhard Med* services are provisioned programmatically following the principle of Infrastructure as Code.

The underlying middleware implements security hardening and controls following the principle that at least 2 levels of security need to be broken before a non-authorized user gains access to data or services. For this reason, the middleware programmatic APIs are strictly restricted to SIS System administrators. API endpoints are not available to users inside *Leonhard Med* tenants. The same security principle is applied to different *Leonhard Med* user services like the user-managed Kubernetes cluster service.

### 5.3 Data transport security

Confidential and strictly confidential data must be transferred to and from *Leonhard Med* using two levels of encryption: an encrypted data transfer protocol and a user level encryption of the data. *Leonhard Med* provides standard services for end-users to encrypt and transfer confidential and strictly confidential data.

Exporting confidential and strictly confidential data from *Leonhard Med* is controlled and under the responsibility of the projects’ Project Leader. Data must be encrypted prior to exporting.

All internal data transport within the Services is secured by Transport Layer Security (TLS). This means that all communication within the services are using HTTPS over TLS communication and hence, use strong encryption for the communication channel.

*Leonhard Med* has different perimeter layers to further control the network access to the user services. Each perimeter is controlled by firewalls, and, where applicable, secure gateways to block unused protocols and services. Traffic from outside, and between, of the services, is programmatically configured to the minimum required in order to provide the services for the users. Network isolation is applied to segregate communication between the application, storage, database, and management tiers.

In case of specific requirements, other non-standard secure data transfer tools and protocols (e.g. Globus [22]) can be deployed and enabled by the *Leonhard Med* team.

### 5.4 Data storage security

The storage infrastructure is hosted at the ETH Zurich datacentre and exclusively operated by selected system administrators of SIS. Operation on confidential and strictly confidential data are monitored and logged in compliance with the Swiss Federal Data Protection act [1]. Logs are the securely stored for 1 year. Data needed for providing the *Leonhard Med* services, such as configurations, logs, and encryption keys, are protected with various methods, such as access control lists (ACLs), segmentation, and encryption.

### 5.5 Data backup and restore

As per the *Leonhard Med* SLA, data storage file systems in *Leonhard Med* are backed up several times per week using the “Backup and Restore” service of ETH. The users’ home directories are backed up to a tape library every night. The backup retention time is 90 days. All *Leonhard Med* backups are encrypted prior to being sent to the tape library and are geo-redundant (two copies in two different locations). The daily backup is automatically monitored, system administrators are notified by e-mail about incidents or failures of the backup system.

### 5.6 Data computing security

The computing environment provided for processing data is an isolated Slurm [20] batch system dedicated to the tenant. Each one can use an own Slurm virtual cluster solely composed of the resources provided to the tenant. Batch and interactive data analysis tasks can be scheduled as Slurm jobs and executed on one of the tenant’s compute or GPU nodes.

### 5.7 Data access security

All users, including the Project Leaders, must sign an agreement stating that they accept and comply with the *Leonhard Med* AUP. There are two access modes provided: web-based and Secure Shell (SSH)-based terminal. Both access modes are regulated by a multi-factor authentication (2FA/MFA) process. Access is possible only from trusted networks such as ETH network, VPN or IP addresses from the allow list.

User access to *Leonhard Med* is monitored and logged. This means that user actions regarding logging in to and out as well as any transfers of user content are logged. Also user actions regarding processing of data through the provided Slurm cluster are logged. Administrative access to the Services is controlled and logged according to ETH Zurich administration guidelines and is limited to select SIS administrators only.

### 5.8 Intrusion detection and prevention security

*Leonhard Med* uses modern security information and event management (SIEM) technology to monitor any attempt to compromise the confidentiality, the integrity, and the availability of the stored data and of the provided services. The services are being regularly scanned for vulnerabilities and other weaknesses. Security patches are applied regularly.

### 5.9 Security assessments

*Leonhard Med* infrastructure and services are subject to security assessments, risk assessments and audits done by internal staff as well as external organizations. The assessments may be global for the whole platform or specific to newly introduced components.

## 6 Software availability

*Leonhard Med* provides a wide array of software which is centrally installed and managed. It includes standard software stacks of Compute Canada (Digital Research Alliance of Canada, [23]) in the form of modules and BioContainers (Galaxy Depot) in the form of Singularity containers.

Ready-to-use software includes also: python and Jupyter notebooks, R, Matlab, Stata, GraphDB. Additional python packages can be installed via conda and pip. Similarly additional R libraries can be locally installed by the users. Those sources are available read-only to all the users via a centrally managed repository manager. *Leonhard Med* provides also development environments such as PyCharm, RStudio and VS Code, available typically via *Leonhard Med* OnDemand secure remote desktop. Also GPU-accelerated deep learning frameworks (such as PyTorch, TensorFlow, Keras, Caffe2) are centrally available.

To install any other software, users have a dedicatated internal application that allow to install software in a controlled way into a specific directory, and finally access the software on *Leonhard Med*. In a similar way, the software can also be installed from public or private repositories on Gitlab or Github instances.

## 7 *Leonhard Med* in action from the user perspective

**Figure 3:**
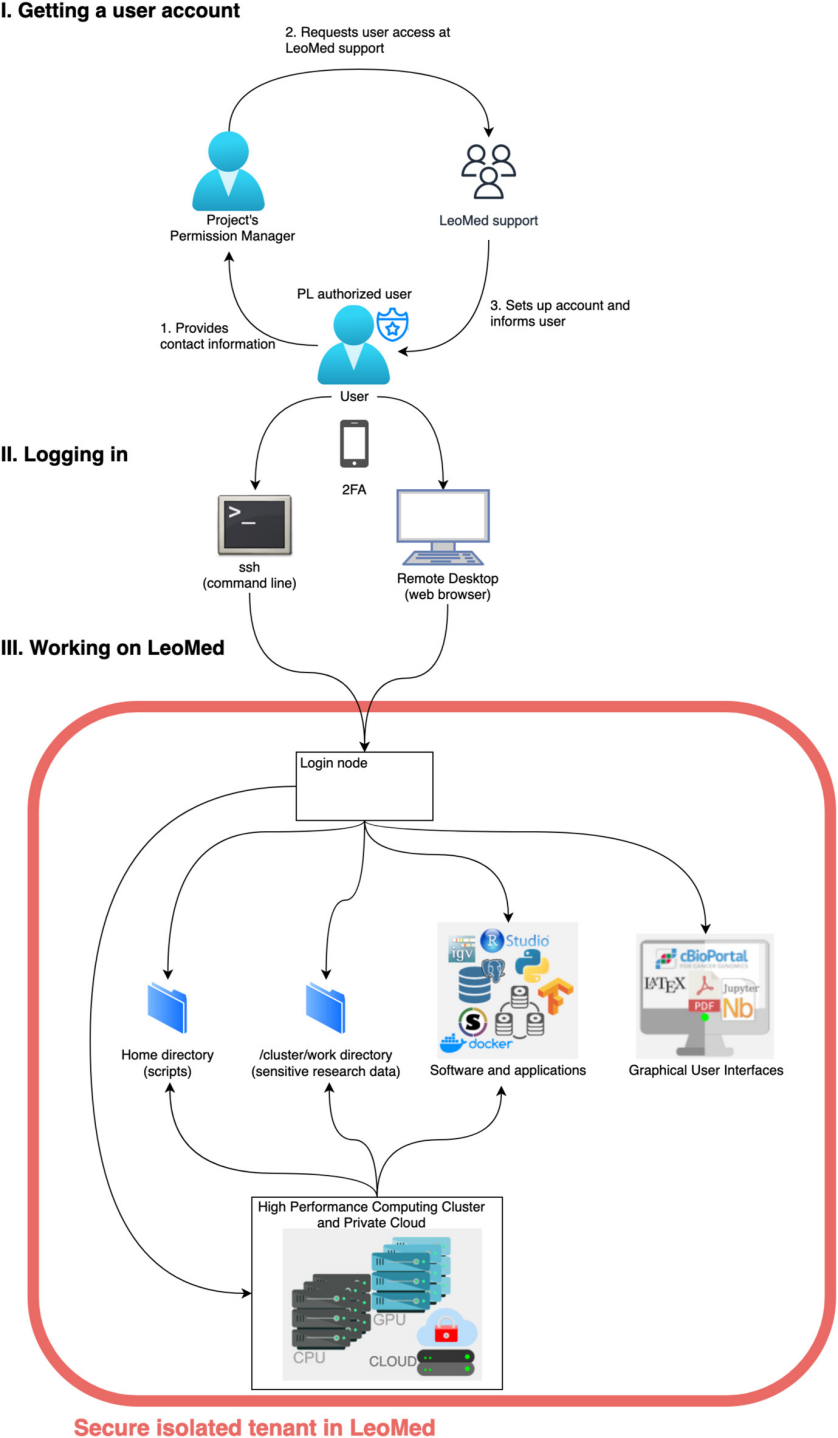
The schema of major user activities: I. getting a user account with the authorization of a permission manager and *Leonhard Med* support, II. logging into *Leonhard Med* with 2FA, III. work in a secure isolated tenant.

A *Leonhard Med* user is an individual who possesses the credentials to access at least one tenant on the TRE. Each secure isolated tenant has one Project Leader (PL) assigned. The PL is responsible for the legal and ethical handling of all strictly confidential and confidential data within a research project. Additionally, the PL is required to request access for users within their respective tenant. The PL also has the option to delegate permission management to a dedicated Permission Manager (PM). All *Leonhard Med* users must agree to the *Leonhard Med* Acceptable Use Policy (AUP) [18] before user access can be requested by the PL or PM. A user account will then be created by the *Leonhard Med* operations team. Authentication for all access to *Leonhard Med* requires 2FA, as illustrated in Figure 3. Users can connect to *Leonhard Med* from authorized networks either via SSH or via the Remote Desktop solution.

*Leonhard Med* implements OnDemand as a remote desktop solution called *Leonhard Med* OnDemand, which provides a comprehensive interactive experience with the respective *Leonhard Med* tenant through a local web browser. Using *Leonhard Med* OnDemand the users can access: remote desktop, shell terminal, interactive data analysis applications (e.g. JupyterLab, RStudio), interactive slurm job submission, upload and download of non-sensitive data.

To support users in working on *Leonhard Med*, the team maintains detailed user documentation which is classified as internal, meaning only users of *Leonhard Med* have access to these resources. In addition, via the *Leonhard Med* service desk, the *Leonhard Med* team reacts to specific questions and requests of *Leonhard Med* users during working hours.

## 8 Platform administration and service operation

The *Leonhard Med* team performs various administration tasks related to user management and ensuring security described in the Section 5, but also a number of operational services. Those are guided by a quality assurance good practices in IT service management, coming from frameworks such as FitSM [24].

### 8.1 Incident and service request management

Incidents and service requests, that are not related to information security incidents, are handled according to a standardized process covering the following: incident detection or service request acknowledgment and case recording, classification, escalation if relevant, incident resolving, and case closing.

### 8.2 Configuration management

All productive servers of *Leonhard Med* are deployed as software code following the Infrastructure as a Code (IaC) process. This allows managing and provisioning the resources through machine-readable definition files, rather than physical hardware configuration.

### 8.3 Change management

Changes in the system configuration are tracked as git commit operations. Each system configuration change includes the timestamp of the change, the name of the system administrator that implemented it, the list of the repository files that have been changed and the details of the changes and the comment describing the rationale.

### 8.4 Release and deployment management

Releases are scheduled in maintenance windows visible in a calendar shared with the users so that they are aware of planned downtimes. Unplanned maintenance and security updates are announced as early as possible. Before deploying updates or new services, releases are tested in a tenant with similar characteristics, to prevent incompatibility and problems for users.

## 9 Conclusions

This paper presents an overview of *Leonhard Med*, a Trusted Research Environment (TRE) tailored for handling sensitive data, particularly within the realm of personalized medicine. Constructing such a platform entails significant investments yet it addresses a pressing requirement within the research community. By meeting this demand, the availability of such a platform can foster heightened research activities involving sensitive data. Once users are assured of a platform that meets legal prerequisites and is user-friendly, they embark on diverse novel data collection endeavors and computational experiments, thereby solidifying the TRE’s role as a crucial asset for the research community.

Additionally, this paper endeavors to highlight key aspects and lessons learned in the development of such a TRE platform. We have built a platform which is secure on all the layers from bare metal, through virtualization to user-driven application layer. At the same time such system must be suitable and friendly according to the needs of the academic and medical user community.

Commencing with user requirements, expertise, and hardware investments, this constantly evolving process necessitates ongoing operational support from a proficient team. This team ensures the consistent quality of service across various domains, including security, documentation, training, adaptability, and issue resolution. Although the endeavor is undeniably long-term and intricate, the current utilization of *Leonhard Med* demonstrates that the effort has been worth investing.
